# Expression of Lipid Metabolism-Related Proteins Differs between Invasive Lobular Carcinoma and Invasive Ductal Carcinoma

**DOI:** 10.3390/ijms18010232

**Published:** 2017-01-23

**Authors:** Yoon Jin Cha, Hye Min Kim, Ja Seung Koo

**Affiliations:** Department of Pathology, Yonsei University College of Medicine, 50-1 Yonsei-ro, Seodaemun-gu, Seoul 03722, Korea; yooncha@yuhs.ac (Y.J.C.); pinkmin15@yuhs.ac (H.M.K.)

**Keywords:** breast cancer, invasive lobular carcinoma, invasive ductal carcinoma, lipid metabolism

## Abstract

We comparatively investigated the expression and clinical implications of lipid metabolism-related proteins in invasive lobular carcinoma (ILC) and invasive ductal carcinoma (IDC) of the breast. A total of 584 breast cancers (108 ILC and 476 IDC) were subjected to tissue microarray and immunohistochemical analysis for lipid metabolism-related proteins including hormone-sensitive lipase (HSL), perilipin A, fatty acid binding protein (FABP)4, carnitine palmitoyltransferase (CPT)-1, acyl-CoA oxidase 1, and fatty acid synthetase (FASN). HSL, perilipin A, and FABP4 expression (all *p* < 0.001) differed significantly: HSL and FABP4 were more frequently present in ILC, whereas perilipin A was more frequently detected in IDC. Among all invasive cancers, HSL and FABP4 were highly expressed in luminal A-type ILC (*p* < 0.001) and perilipin A in luminal A-type IDC (*p* = 0.007). Among luminal B-type cancers, HSL and FABP4 were more highly expressed in ILC (*p* < 0.001). Univariate analysis found associations of shorter disease-free survival with CPT-1 positivity (*p* = 0.004) and acyl-CoA oxidase 1 positivity (*p* = 0.032) and of shorter overall survival with acyl-CoA oxidase 1 positivity (*p* = 0.027). In conclusion, ILC and IDC exhibited different immunohistochemical lipid metabolism-related protein expression profiles. Notably, ILC exhibited high HSL and FABP4 and low perilipin A expression.

## 1. Introduction

Invasive breast cancer, the most common type of cancer affecting women, can be roughly subdivided into invasive ductal carcinoma (IDC) and invasive lobular carcinoma (ILC) [1]. ILC comprises approximately 5%–15% of all cases of invasive carcinoma [2,3], although hormone replacement therapy and increased alcohol consumption have led recently to a more rapid increase in the incidence of ILC than of IDC [4,5]. Compared to IDC, ILC more frequently presents as multiple and bilateral lesions [6,7], and is histologically characterized by non-cohesive cancer cells lacking e-cadherin expression [8]. Furthermore, ILC commonly metastasizes to the bone, gastrointestinal tract, uterus, meninges, and ovarian diffuse serosal surface, in contrast to the metastasis patterns exhibited by IDC [7,9,10].

The most profound metabolic difference between tumor cells and normal cells can be summarized as the Warburg effect, wherein tumor cells produce energy via aerobic glycolysis rather than aerobic phosphorylation during the tricarboxylic acid cycle [11]. Although glycolysis is an important metabolic process in the cancer cell, these cells exhibit characteristic metabolic flexibility, which may be exploitable by cancer metabolic therapy. Metabolic reprogramming in cancer includes glucose, amino acid and lipid metabolism (such as lipolysis, lipid transfer, and β-oxidation).

Several enzymes function within the lipid metabolism pathway. For example, hormone-sensitive lipase (HSL) hydrolyzes triglycerides into free fatty acids [12], whereas during lipolysis, perilipin A acts as a lipid droplet gate-keeper [13] and fatty acid binding protein (FABP) plays key roles in lipid transfer and functions as a free fatty acid transporter [14]. Additionally, carnitine palmitoyltransferase 1 (CPT-1) [15] and acyl-CoA oxidase 1 [16] are essential enzymes in the process of β-oxidation. Previous comparative genomic analyses have demonstrated different mechanisms of lipid/fatty acid transport and metabolism in ILC and IDC [17], with high expression of lipid biosynthesis genes in the former [18]. Another previous study reported the differential expression of metabolism-related proteins between ILC and IDC [19], suggesting that these types of cancer may exhibit different patterns of lipid metabolism-related protein expression. In the present study, therefore, we aimed to evaluate the expression and clinical implications of lipid metabolism-related proteins in ILC.

## 2. Results

### 2.1. Basal Characteristics of ILC and IDC

The present study included 97 (89.8%) classic-type and 11 (11.1%) pleomorphic-type ILCs; the latter were significantly associated with an older patient age (*p* = 0.011), higher nuclear grade (*p* < 0.001), higher histologic grade (*p* < 0.001), higher pathologic tumor stage (*p* = 0.048), progesterone receptor (PR) negativity (*p* = 0.018), human epidermal growth factor receptor 2 (HER-2) positivity (*p* = 0.002), higher Ki-67 labeleing index (LI) (*p* = 0.001), and non-luminal A subtype (*p* < 0.001) when compared with the former. The clinicopathologic characteristics of ILC are summarized in Table S1. Basal characteristics of the 476 IDCs included for comparison are summarized in Table S2.

### 2.2. Expression of Lipid Metabolism-Related Proteins in ILC According to Histologic Type

**Figure 1:**
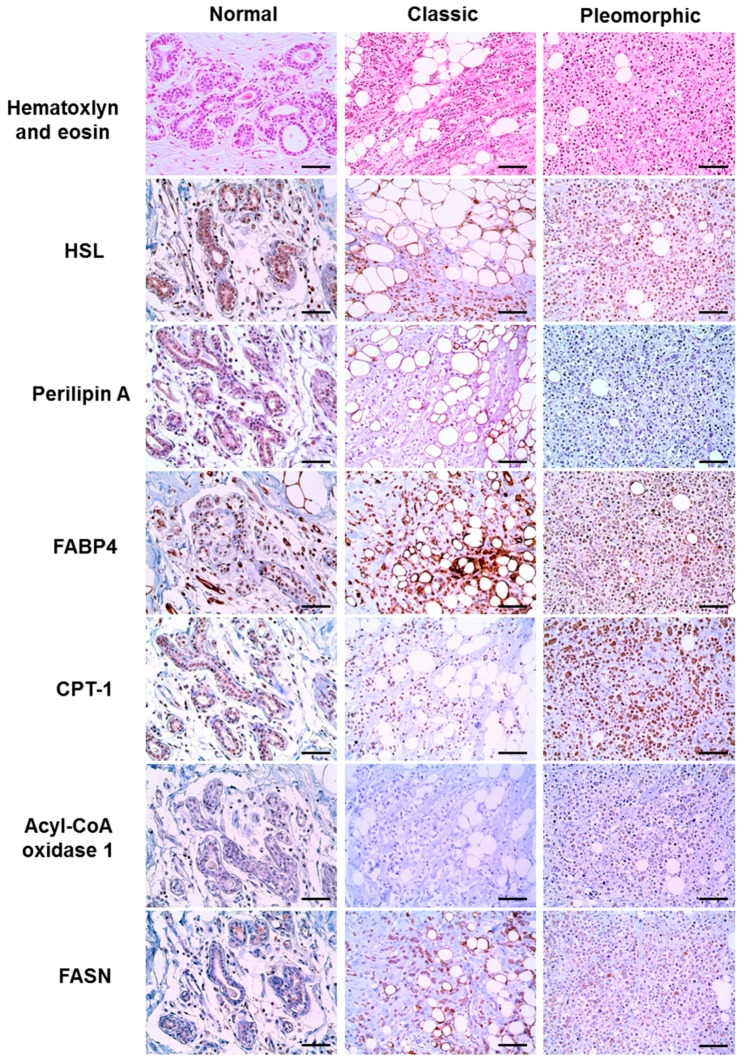
Expression of lipid metabolism-related proteins in invasive lobular carcinoma (ILC) according to histologic type. Higher expression levels of carnitine palmitoyltransferase 1 (CPT-1) and acyl-CoA oxidase 1 are observed in pleomorphic-type ILC when compared to classic-type ILC. Nearby normal breast tissue shows reduced presence or absence of expression of lipid metabolism-related proteins compared to the tumor cells. Scale bar = 100 μm.

In an evaluation according to ILC histologic type, CPT-1 and acyl-CoA oxidase 1 were more highly expressed in pleomorphic-type tumors (*p* = 0.029 and *p* = 0.014, respectively), whereas perilipin A expression was absent in tumor cells (Figure 1 and Table 1). Nearby normal breast tissue revealed reduced presence or absence of lipid metabolism-related proteins compared to the tumor cells (Figure 1).

### 2.3. Comparison of the Expression of Lipid Metabolism-Related Proteins between ILC and IDC

**Figure 2:**
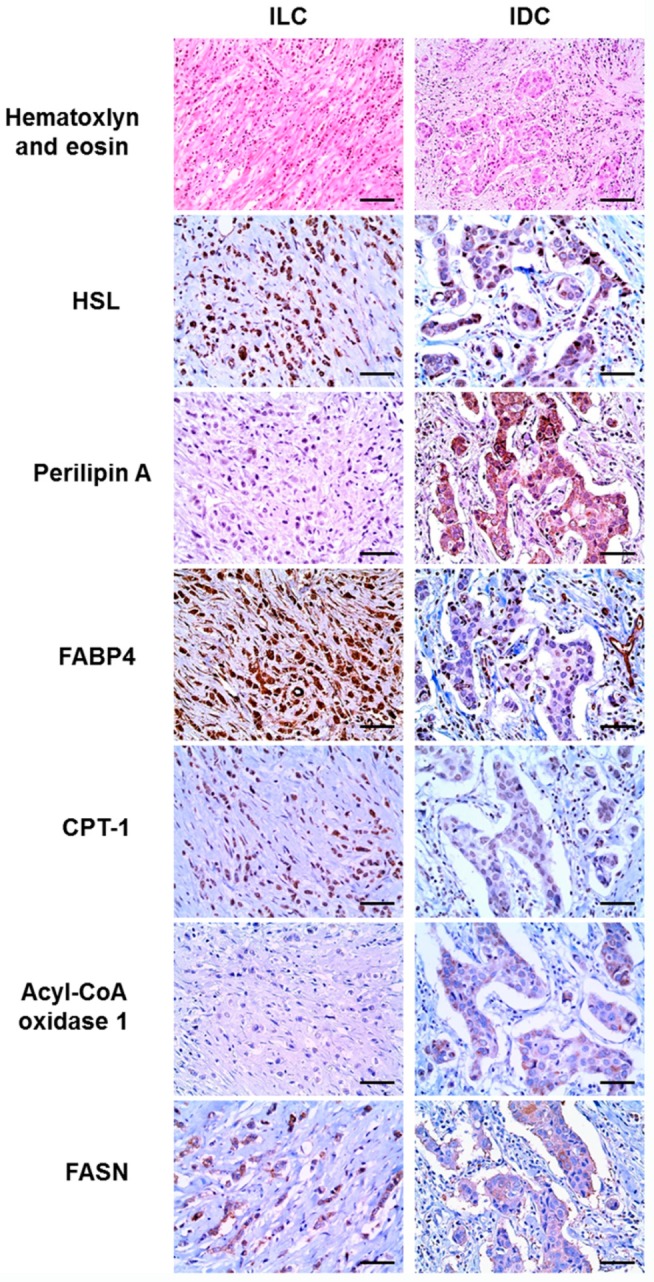
Expression of lipid metabolism-related proteins in invasive lobular carcinoma (ILC) and invasive ductal carcinoma (IDC). Higher expression levels of hormone-sensitive lipase (HSL) and fatty acid binding protein 4 (FABP4) are observed in invasive lobular carcinoma, whereas higher levels of perilipin A are observed in invasive ductal carcinoma. Scale bar = 100 μm.

Differential expression of lipid metabolism-related proteins was observed in ILC and IDC (Table 2). HSL (*p* < 0.001) and FABP4 (*p* < 0.001) showed higher expression rates in ILC, whereas perilipin A (*p* < 0.001) was more highly expressed in IDC (Figure 2). Because most ILC cases included in this study were of the luminal type, we further analyzed the expression of lipid metabolism-related proteins in luminal-type IDCs and ILCs to determine whether the previously observed differential expression of lipid metabolism-related proteins was molecular subtype-dependent (Table 3). Among luminal A-type tumors, ILC exhibited higher expression of HSL (*p* < 0.001) and FABP4 (*p* < 0.001), whereas IDC exhibited higher expression of perilipin A (*p* = 0.007). Similarly, ILC luminal B-type tumors more highly expressed HSL (*p* < 0.001) and FABP4 (*p* < 0.001), compared with IDC.

### 2.4. Correlation between Lipid Metabolism Proteins and Clinicopathologic Factors in ILC

**Figure 3:**
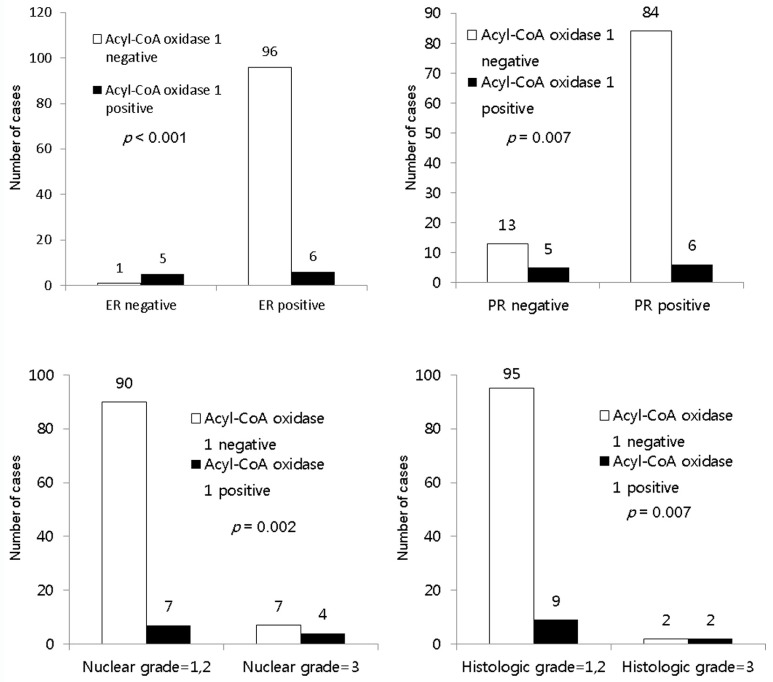
Correlation between the expression of lipid metabolism-related proteins and clinicopathologic factors in invasive lobular carcinoma. In ILC, acyl-CoA oxidase 1 expression is associated with ER negativity (*p* < 0.001), PR negativity (*p* = 0.007), higher nuclear grade (*p* = 0.002), and higher histologic grade (*p* = 0.007).

In ILC, acyl-CoA oxidase 1 expression was associated with estrogen receptor (ER) negativity (*p* < 0.001), PR negativity (*p* = 0.007), a higher nuclear grade (*p* = 0.002), and higher histologic grade (*p* = 0.007) (Figure 3).

### 2.5. Impact of Lipid Metabolism-Related Protein Expression on Prognosis in ILC

**Figure 4:**
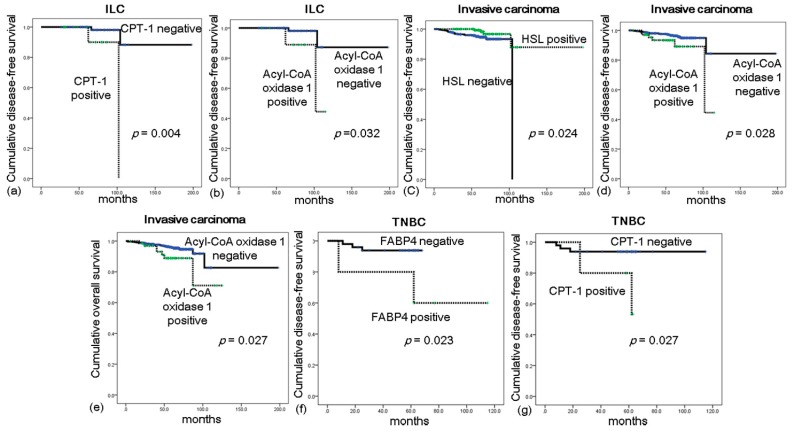
Impact of lipid metabolism-related protein expression on prognosis. In invasive lobular carcinoma, shorter disease-free survival (DFS) is shown to associate with carnitine palmitoyltransferase 1 (CPT-1) positivity (**a**) and acyl-CoA oxidase 1 positivity (**b**); Among all invasive breast cancers, a shorter DFS is associated with hormone-sensitive lipase (HSL) negativity (**c**) and acyl-CoA oxidase 1 positivity (**d**); whereas shorter overall survival (OS) is associated only with acyl-CoA oxidase 1 positivity (**e**); Among triple-negative breast cancers (TNBCs), a shorter DFS is associated with fatty acid binding protein 4 (FABP4) positivity (**f**) and CPT-1 positivity (**g**).

In a univariate analysis of ILC, CPT-1 positivity (*p* = 0.004) and acyl-CoA oxidase 1 positivity (*p* = 0.032) were associated with a shorter disease-free survival (DFS; Table 4 and Figure 4); however, no significantly predictive protein expression status was identified in a multivariate analysis (Table S3). In a univariate analysis of all invasive breast cancers (*n* = 584), a shorter DFS was associated with HSL negativity (*p* = 0.024) and acyl-CoA oxidase 1 positivity (*p* = 0.028), whereas a shorter overall survival (OS) was associated only with acyl-CoA oxidase 1 positivity (*p* = 0.027) (Table 5). In a prognostic analysis of all invasive breast cancers according to molecular subtype, FABP4 positivity (*p* = 0.023) and CPT-1 positivity (*p* = 0.027) were associated with a shorter DFS among triple negative breast cancers (TNBCs) (Figure 4).

## 3. Discussion

This investigation of lipid metabolism-related protein expression in ILC and IDC revealed characteristic patterns involving higher expression of HSL and FABP4 in ILC and perilipin A in IDC. A previous hierarchical clustering study showed four distinct groups of breast cancer, based on the gene expression level [17]. In that study, group IV, which was predominantly comprised of ILC, exhibited very high levels of adipose tissue marker expression relative to the other groups [17]. In addition, ILCs classified into group II also exhibited high levels of adipose tissue marker expression [17], findings consistent with the results of present study. That study also found high expression of the adipose tissue markers FABP4 and lipase in ILC [17], as demonstrated in the present study. Our study and the previous study differ, however, with regard to perilipin expression; the previous study reported upregulation of this protein in ILC [17], whereas our study failed to detect this protein in ILC tumor cells. We attribute this discrepancy to the evaluation of different cell compartments. The previous study used whole-tissue profiling, in which average gene expression level from all cells in a tumor sample is evaluated regardless of cell type. Meanwhile, we used immunohistochemistry to discriminate specific expression sites (e.g., tumor or stromal cells) of each molecule. Using this method, we observed perilipin A expression in adipocytes within the tumor stroma of ILC, and note that further study is needed to identify the cellular origin of this expression. We further confirmed the differential expression of lipid metabolism-related proteins in ILC and IDC samples was restricted to luminal-type tumors, suggesting that the observed differences in lipid metabolism-related protein expression are independent of molecular subtype. This finding was also observed by Weigelt et al., who found that the different transcriptomes of ILC and IDC were retained even after molecular subtype matching [18].

In the present study, we observed different levels of lipid metabolism-related protein expression between the two ILC subtypes; specifically, pleomorphic-type tumors showed higher expression of CPT-1 and acyl-CoA oxidase 1. The pleomorphic-type ILC is an aggressive variant known to harbor more adverse biomarker profiles such as hormone receptor negativity, HER-2 positivity, and a high Ki-67 LI when compared with classic-type ILC [20,21]. Despite differences in tumor biology, however, molecular studies have revealed a common molecular genetic pathway shared by pleomorphic- and classic-type tumors [22,23,24]. A previous finding of higher perilipin expression in classic-type ILC vs. ductal-like ILC [17] suggested the potential for differential expression of adipocyte-related molecules in ILC subgroups. Aberrant CPT-1 expression has been found to be associate with high-grade glioma [25], suggesting a correlation between lipid metabolism-related protein expression and a higher tumor grade. In ILC, CPT-1 positivity and acyl-CoA oxidase 1 positivity were found to correlate with poor prognosis. This finding was concordant with previous studies that observed associations between poor prognosis and CPT-1 expression in esophageal cancer [26] or acyl-CoA oxidase 1 positivity in breast cancer [27]. We also found the association between expression of lipid metabolism-related proteins and poor prognosis in the breast cancer subtypes: HSL negativity and acyl-CoA oxidase 1 positivity in invasive breast cancer, and FABP4 positivity and CPT-1 positivity in the TNBC subgroup. Thus, lipid metabolism-related proteins appear to be a potential prognostic factor in variable subgroups of breast cancer, reflecting tumor aggressiveness.

In addition to the upregulated adipose markers in ILC [17], the inhibition of FABP-4 [28] and CPT-1 [29,30], which were found to be highly expressed in ILC and pleomorphic-type ILC, respectively, have been reported to block tumor growth, indicating that lipid metabolism could be targeted in the context of ILC treatment. Increase of fat oxidation and subsequent lipolysis are observed under exercise of low to moderate intensity, resulted by increased fatty acid availability. However, lipolysis is reduced at high exercise intensity due to increase of carbohydrate oxidation from glycolytic flux and reduced CPT-1 activity [31]. This phenomenon has clinical relevance that could reduce the insulin resistance and metabolic disease, which can increase risk of cancer development, including breast cancer [32]. As described in present study, CPT-1 is a potential therapeutic target in ILC, particularly in the pleomorphic subtype, and indirect reduction of CPT-1 activity via intense exercise may have a therapeutic effect in ILC patients. Since obesity is one risk factor associated with poor outcome of breast cancer patients [33], exercise could improve indirectly also the prognosis in women with breast cancer [32,34].

One of major limitation of present study was an unbalanced patient number among the subgroups. There were relatively small numbers of ILC patients when compared to IDC, and even fewer numbers of pleomorphic-type ILC patients in comparison to classic-type ILC, which might be inappropriate for statistical analysis, and could lead to skewed results. However, this imbalance is derived from its small intrinsic prevalence of ILC and pleomorphic-type ILC. In prior studies, when comparing IDC and ILC, ILC represented approximately 10% in proportion [35,36,37]. Furthermore, pleomorphic-type ILC accounts for 13% of ILC cases; this proportion could be even smaller in the overall invasive breast cancer category [38].

In conclusion, we observed different expression profiles of lipid metabolism-related proteins in ILC when compared with IDC. ILC showed higher expression of HSL and FABP4 and lower expression of perilipin A. In addition, CPT-1 and acyl-CoA oxidase 1 expression were found to be associated with a shorter DFS in ILC. Discovery of the differential expression lipid metabolism-related proteins in ILC has clinical implications, as it could provide potential therapeutic targets.

## 4. Materials and Methods

### 4.1. Patient Selection and Clinicopathologic Evaluation

This study was approved by the Institutional Review Board (IRB) of Severance Hospital (IRB No. 4-2014-0701, October 2014). The informed consent form patient was waivered by the IRB. The study was conducted in accordance with the Declaration of Helsinki. From January 2000 to December 2012; a total of 108 patients who were diagnosed with ILC and underwent surgical resection at Severance Hospital were selected. An additional 476 patients with IDC of no specific type were included for comparison. Patients who had received neoadjuvant chemotherapy were excluded.

For the histologic analysis, all hematoxylin and eosin (H&E)-stained slides were retrospectively reviewed by a breast pathologist (Koo, J.S.), who assessed the histological grade according to the Nottingham grading system [39]. Tumor staging was based on the seventh American Joint Committee on Cancer (AJCC) criteria. Disease-free survival (DFS) was calculated from the date of the first curative surgery to the date of the first loco-regional or systemic relapse, or death without any type of relapse. Overall survival (OS) was estimated from the date of the first curative operation to the date of the last follow-up or death from any cause. The clinicopathologic parameters evaluated for each breast cancer included the patient’s age at initial diagnosis, lymph node metastasis, tumor recurrence, distant metastasis, and survival status.

### 4.2. Tissue Microarray

After a review of H&E-stained slides, matched formalin-fixed paraffin-embedded (FFPE) tumor tissue samples were retrieved for the most appropriate sections. Subsequently, tissue microarrays were constructed using two 3-mm tissue cores punched from each retrieved FFPE tumor tissue. The most representative tumor areas were selected from each tumor.

### 4.3. Immunohistochemistry

Antibodies used for immunohistochemistry in this study are shown in Table 6. Immunohistochemical staining was applied to FFPE tissue sections. Briefly, 3-mm paraffin sections were deparaffinized and rehydrated in solutions of xylene and alcohol. Immunohistochemistry was performed using a Ventana Discovery XT automated stainer (Ventana Medical Systems, Tucson, AZ, USA), following antigen retrieval with Cell Conditioning 1 (CC1; citrate buffer pH 6.0, Ventana Medical System). Appropriate positive and negative controls for immunohistochemistry were included. 

### 4.4. Interpretation of Immunohistochemical Results

A cut-off value of ≥1% positively-stained nuclei was used to define estrogen receptor (ER) and progesterone receptor (PR) positivity [40]. Human epidermal growth factor receptor 2 (HER-2) staining was analyzed according to the American Society of Clinical Oncology (ASCO)/College of American Pathologists (CAP) guidelines, using the following categories: 0 = no immunostaining; 1+ = weak incomplete membranous staining, <10% of tumor cells; 2+ = complete membranous staining, either uniform or weak in ≥ 10% of tumor cells; and 3+ = uniform intense membranous staining in ≥30% of tumor cells [41]. HER-2 expression was considered positive when strong (3+) membranous staining was observed, whereas cases with scores of 0 to 1+ were considered negative. Cases exhibiting equivocal (2+) HER-2 expression were subjected to further evaluation of HER-2 gene amplification using fluorescent in situ hybridization (FISH).

The results of immunohistochemical staining for lipid metabolism-related proteins were scored by multiplying scores indicating the proportion of stained cells (negative, 0; <30% positive, 1; ≥30% positive, 2) by scores indicating the immunostaining intensity (negative, 0; weak, 1; moderate, 2; strong, 3). Scores of 0–1 were interpreted as negative; scores of 2–6 were considered positive [42].

### 4.5. Tumor Phenotype Classification

In this study, we classified breast cancer phenotypes according to ER, PR, and HER-2 immunohistochemistry results and Ki-67 labeling index (LI). HER-2 FISH results were used to categorize tumors as follows [43]: luminal A type: ER and/or PR positive, HER-2 negative, and Ki-67 LI < 14%; luminal B type: (HER-2 negative) ER and/or PR positive, HER-2 negative, and Ki-67 LI ≥ 14% and (HER-2 positive) ER and/or PR positive and HER-2 overexpressed and/or amplified; HER-2 type: ER and PR negative and HER-2 overexpressed and/or amplified; TNBC (triple-negative breast cancer) type: ER, PR, and HER-2 negative.

### 4.6. Statistical Analysis

Data were statistically processed using SPSS for Windows, version 20.0 (SPSS Inc., Chicago, IL, USA). Student’s *t*-test and Fisher’s exact test were used to assess continuous and categorical variables, respectively. Statistical significance was assumed at a *p*-value < 0.05. Kaplan–Meier survival curves and log-rank statistics were employed to evaluate the time interval to tumor metastasis and survival duration. A Cox proportional hazards model was used to assess the risk factors of shorter DFS and OS.

## Figures and Tables

**Table 1:** Expression of lipid metabolism-related proteins in ILC according to histologic type.

Parameters	Total *n* = 108 (%)	Classic Type *n* = 97 (%)	Pleomorphic Type *n* = 11 (%)	*p*-Value
HSL				0.589
Negative	8 (7.4)	7 (7.2)	1 (9.1)	
Positive	100 (92.6)	90 (92.8)	10 (90.9)	
Perilipin A				N/A
Negative	108 (100.0)	97 (100.0)	11 (100.0)	
Positive	0 (0.0)	0 (0.0)	0 (0.0)	
FABP4				1.000
Negative	73 (67.6)	65 (67.0)	8 (72.7)	
Positive	35 (32.4)	32 (33.0)	3 (27.3)	
CPT-1				**0.029**
Negative	88 (81.5)	82 (84.5)	6 (54.5)	
Positive	20 (18.5)	15 (15.5)	5 (45.5)	
Acyl-CoA oxidase 1				**0.014**
Negative	97 (89.8)	90 (92.8)	7 (63.6)	
Positive	11 (10.2)	7 (7.2)	4 (36.4)	
FASN				0.748
Negative	72 (66.7)	64 (66.0)	8 (72.7)	
Positive	36 (33.3)	33 (34.0)	3 (27.3)	

N/A, not applicable; HSL, hormone-sensitive lipase; FABP4, fatty acid binding protein 4; CPT-1, carnitine palmitoyltransferase 1; FASN, fatty acid synthetase. Significant values in bold.

**Table 2:** Expression of lipid metabolism-related proteins in ILC and IDC.

Parameters	Total *n* = 584 (%)	ILC *n* = 108 (%)	IDC *n* = 476 (%)	*p*-Value
HSL				**<0.001**
Negative	414 (70.9)	8 (7.4)	406 (85.3)	
Positive	170 (29.1)	100 (92.6)	70 (14.7)	
Perilipin A				**<0.001**
Negative	530 (90.8)	108 (100.0)	422 (88.7)	
Positive	54 (9.2)	0 (0.0)	54 (11.3)	
FABP4				**<0.001**
Negative	541 (92.6)	73 (67.6)	468 (98.3)	
Positive	43 (7.4)	35 (32.4)	8 (1.7)	
CPT-1				0.322
Negative	494 (84.6)	88 (81.5)	406 (85.3)	
Positive	90 (15.4)	20 (18.5)	70 (14.7)	
Acyl-CoA oxidase 1				0.561
Negative	515 (88.2)	97 (89.8)	418 (87.8)	
Positive	69 (11.8)	11 (10.2)	58 (12.2)	
FASN				0.825
Negative	384 (65.8)	72 (66.7)	312 (65.5)	
Positive	200 (34.2)	36 (33.3)	164 (34.5)	

ILC, invasive lobular carcinoma; IDC, invasive ductal carcinoma; HSL, hormone-sensitive lipase; FABP4, fatty acid binding protein 4; CPT-1, carnitine palmitoyltransferase 1; FASN, fatty acid synthetase. Significant values in bold.

**Table 3:** Comparison of lipid metabolism-related protein expression in luminal-type ILC and IDC.

Parameters	Luminal A Type			Luminal B Type		
	ILC *n* = 82 (%)	IDC *n* = 242 (%)	*p*-Value	ILC *n* = 21 (%)	IDC *n* = 134 (%)	*p*-Value
HSL			**<0.001**			**<0.001**
Negative	5 (6.1)	199 (82.2)		3 (14.3)	115 (85.8)	
Positive	77 (93.9)	43 (17.8)		18 (85.7)	19 (14.2)	
Perilipin A			**0.007**			0.120
Negative	82 (100.0)	222 (91.7)		21 (100.0)	120 (89.6)	
Positive	0 (0.0)	20 (8.3)		0 (0.0)	14 (10.4)	
FABP4			**<0.001**			**<0.001**
Negative	57 (69.5)	240 (99.2)		14 (66.7)	133 (99.3)	
Positive	25 (30.5)	2 (0.8)		7 (33.3)	1 (0.7)	
CPT-1			0.442			0.175
Negative	71 (86.6)	217 (89.7)		14 (66.7)	107 (79.9)	
Positive	11 (13.4)	25 (10.3)		7 (33.3)	27 (20.1)	
Acyl-CoA oxidase 1			0.515			0.521
Negative	78 (95.1)	234 (96.7)		18 (85.7)	121 (90.3)	
Positive	4 (4.9)	8 (3.3)		3 (14.3)	13 (9.7)	
FASN			0.657			0.340
Negative	52 (63.4)	160 (66.1)		16 (76.2)	88 (65.7)	
Positive	30 (36.6)	82 (33.9)		5 (23.8)	46 (34.3)	

ILC, invasive lobular carcinoma; IDC, invasive ductal carcinoma; HSL, hormone-sensitive lipase; FABP4, fatty acid binding protein 4; CPT-1, carnitine palmitoyltransferase 1; FASN, fatty acid synthetase. Significant values in bold.

**Table 4:** Univariate analysis (log-rank test) of the impacts of lipid metabolism-related protein expression in invasive lobular carcinoma on disease-free and overall survival.

Parameters	Disease-Free Survival		Overall Survival	
	95% CI	*p*-Value	95% CI	*p*-Value
HSL		0.286		N/A
Negative	104 (104–104)		N/A	
Positive	187 (167–201)		N/A	
FABP4		0.573		0.326
Negative	177 (154–200)		183 (165–200)	
Positive	111 (104–118)		110 (100–120)	
CPT-1		**0.004**		N/A
Negative	186 (168–203)		N/A	
Positive	98 (87–108)		N/A	
Acyl-CoA oxidase 1		**0.032**		0.759
Negative	185 (165–204)		181 (162–199)	
Positive	103 (90–115)		117 (104–130)	
FASN		0.150		0.787
Negative	184 (165–204)		178 (158–197)	
Positive	103 (96–110)		122 (116–127)	

CI, confidence interval; HSL, hormone-sensitive lipase; N/A, not applicable; FABP4, fatty acid binding protein 4; CPT-1, carnitine palmitoyltransferase 1; FASN, fatty acid synthetase. Significant values in bold.

**Table 5:** Univariate analysis (log-rank test) of the impacts of lipid metabolism-related protein expression in invasive breast cancers on disease-free and overall survival.

Parameters	Disease-Free Survival		Overall Survival	
	95% CI	*p*-Value	95% CI	*p*-Value
HSL		0.024		0.154
Negative	99 (97–101)		104 (102–106)	
Positive	185 (168–201)		178 (160–195)	
Perillipin A		0.977		0.795
Negative	173 (153–193)		176 (161–190)	
Positive	81 (78-84)		81 (78–84)	
FABP4		0.895		0.607
Negative	172 (150–193)		177 (161–193)	
Positive	109 (101–116)		108 (97–119)	
CPT-1		0.183		0.194
Negative	180 (163–197)		175 (159–192)	
Positive	97 (93–101)		117 (111–122)	
Acyl-CoA oxidase 1		**0.028**		**0.027**
Negative	180 (161–198)		177 (160–195)	
Positive	100 (90–111)		108 (94–122)	
FASN		0.313		0.112
Negative	177 (159–196)		171 (153–189)	
Positive	104 (98–109)		121 (119–124)	

CI, confidence interval; HSL, hormone-sensitive lipase; FABP4, fatty acid binding protein 4; CPT-1, carnitine palmitoyltransferase 1; FASN, fatty acid synthetase. Significant values in bold.

**Table 6:** Source, clones, and dilutions of antibodies used in this study.

Antibody	Company	Clone	Dilution
Lipolysis-related			
HSL	Abcam, Cambridge, UK	Polyclonal	1:100
Perilipin A	Abcam, Cambridge, UK	Polyclonal	1:100
FABP4	Abcam, Cambridge, UK	Polyclonal	1:100
CPT-1	Abcam, Cambridge, UK	8F6AE9	1:200
Acyl-CoA oxidase 1	Abcam, Cambridge, UK	Polyclonal	1:50
FASN	Abcam, Cambridge, UK	Polyclonal	1:100
Molecular subtype related proteins			
ER	Thermo Scientific, San Siego, CA, USA	SP1	1:100
PR	DAKO, Glostrup, Denmark	PgR	1:50
HER-2	DAKO, Glostrup, Denmark	Polyclonal	1:1500
Ki-67	Abcam, Cambridge, UK	MIB	1:1000

HSL, hormone-sensitive lipase; FABP4, fatty acid binding protein 4; CPT-1, carnitine palmitoyltransferase-1; FASN, fatty acid synthetase; ER, estrogen receptor; PR, progesterone receptor; HER-2, human epidermal growth factor receptor 2.

## Supplementary Materials

Supplementary materials can be found at www.mdpi.com/1422-0067/18/1/232/s1.

## References

[1] Tavassoli F.A., Devilee P., International Agency for Research on Cancer, World Health Organization (2003). Pathology and Genetics of Tumours of the Breast and Female Genital Organs.

[2] Li C.I., Anderson B.O., Daling J.R., Moe R.E. (2003). Trends in incidence rates of invasive lobular and ductal breast carcinoma. JAMA.

[3] Li C.I., Uribe D.J., Daling J.R. (2005). Clinical characteristics of different histologic types of breast cancer. Br. J. Cancer.

[4] Li C.I., Chlebowski R.T., Freiberg M., Johnson K.C., Kuller L., Lane D., Lessin L., O’Sullivan M.J., Wactawski-Wende J., Yasmeen S. (2010). Alcohol consumption and risk of postmenopausal breast cancer by subtype: The women’s health initiative observational study. J. Natl. Cancer Inst..

[5] Reeves G.K., Beral V., Green J., Gathani T., Bull D. (2006). Hormonal therapy for menopause and breast-cancer risk by histological type: A cohort study and meta-analysis. Lancet Oncol..

[6] Lesser M.L., Rosen P.P., Kinne D.W. (1982). Multicentricity and bilaterality in invasive breast carcinoma. Surgery.

[7] Silverstein M.J., Lewinsky B.S., Waisman J.R., Gierson E.D., Colburn W.J., Senofsky G.M., Gamagami P. (1994). Infiltrating lobular carcinoma. Is it different from infiltrating duct carcinoma?. Cancer.

[8] De Leeuw W.J., Berx G., Vos C.B., Peterse J.L., van de Vijver M.J., Litvinov S., van Roy F., Cornelisse C.J., Cleton-Jansen A.M. (1997). Simultaneous loss of E-cadherin and catenins in invasive lobular breast cancer and lobular carcinoma in situ. J. Pathol..

[9] Sastre-Garau X., Jouve M., Asselain B., Vincent-Salomon A., Beuzeboc P., Dorval T., Durand J.C., Fourquet A., Pouillart P. (1996). Infiltrating lobular carcinoma of the breast. Clinicopathologic analysis of 975 cases with reference to data on conservative therapy and metastatic patterns. Cancer.

[10] Lamovec J., Bracko M. (1991). Metastatic pattern of infiltrating lobular carcinoma of the breast: An autopsy study. J. Surg. Oncol..

[11] Warburg O. (1956). On the origin of cancer cells. Science.

[12] Kraemer F.B., Shen W.J. (2002). Hormone-sensitive lipase: Control of intracellular tri-(di-)acylglycerol and cholesteryl ester hydrolysis. J. Lipid Res..

[13] Greenberg A.S., Egan J.J., Wek S.A., Garty N.B., Blanchette-Mackie E.J., Londos C. (1991). Perilipin, a major hormonally regulated adipocyte-specific phosphoprotein associated with the periphery of lipid storage droplets. J. Biol. Chem..

[14] Weisiger R.A. (2002). Cytosolic fatty acid binding proteins catalyze two distinct steps in intracellular transport of their ligands. Mol. Cell. Biochem..

[15] Bonnefont J.P., Djouadi F., Prip-Buus C., Gobin S., Munnich A., Bastin J. (2004). Carnitine palmitoyltransferases 1 and 2: Biochemical, molecular and medical aspects. Mol. Asp. Med..

[16] Kawaguchi A., Tsubotani S., Seyama Y., Yamakawa T., Osumi T., Hashimoto T., Kikuchi T., Ando M., Okuda S. (1980). Stereochemistry of dehydrogenation catalyzed by acyl-CoA oxidase. J. Biochem..

[17] Zhao H., Langerod A., Ji Y., Nowels K.W., Nesland J.M., Tibshirani R., Bukholm I.K., Karesen R., Botstein D., Borresen-Dale A.L. (2004). Different gene expression patterns in invasive lobular and ductal carcinomas of the breast. Mol. Biol. Cell.

[18] Weigelt B., Geyer F.C., Natrajan R., Lopez-Garcia M.A., Ahmad A.S., Savage K., Kreike B., Reis-Filho J.S. (2010). The molecular underpinning of lobular histological growth pattern: A genome-wide transcriptomic analysis of invasive lobular carcinomas and grade and molecular subtype-matched invasive ductal carcinomas of no special type. J. Pathol..

[19] Kim Y.H., Jung W.H., Koo J.S. (2014). Expression of metabolism-related proteins in invasive lobular carcinoma: Comparison to invasive ductal carcinoma. Tumour Biol..

[20] Jacobs M., Fan F., Tawfik O. (2012). Clinicopathologic and biomarker analysis of invasive pleomorphic lobular carcinoma as compared with invasive classic lobular carcinoma: An experience in our institution and review of the literature. Ann. Diagn. Pathol..

[21] Frolik D., Caduff R., Varga Z. (2001). Pleomorphic lobular carcinoma of the breast: Its cell kinetics, expression of oncogenes and tumour suppressor genes compared with invasive ductal carcinomas and classical infiltrating lobular carcinomas. Histopathology.

[22] Reis-Filho J.S., Simpson P.T., Jones C., Steele D., Mackay A., Iravani M., Fenwick K., Valgeirsson H., Lambros M., Ashworth A. (2005). Pleomorphic lobular carcinoma of the breast: Role of comprehensive molecular pathology in characterization of an entity. J. Pathol..

[23] Simpson P.T., Reis-Filho J.S., Lambros M.B., Jones C., Steele D., Mackay A., Iravani M., Fenwick K., Dexter T., Jones A. (2008). Molecular profiling pleomorphic lobular carcinomas of the breast: Evidence for a common molecular genetic pathway with classic lobular carcinomas. J. Pathol..

[24] Vargas A.C., Lakhani S.R., Simpson P.T. (2009). Pleomorphic lobular carcinoma of the breast: Molecular pathology and clinical impact. Future Oncol..

[25] Cirillo A., Di Salle A., Petillo O., Melone M.A., Grimaldi G., Bellotti A., Torelli G., De’ Santi M.S., Cantatore G., Marinelli A. (2014). High grade glioblastoma is associated with aberrant expression of ZFP57, a protein involved in gene imprinting, and of CPT1A and CPT1C that regulate fatty acid metabolism. Cancer Biol. Ther..

[26] Shi Z.Z., Liang J.W., Zhan T., Wang B.S., Lin D.C., Liu S.G., Hao J.J., Yang H., Zhang Y., Zhan Q.M. (2011). Genomic alterations with impact on survival in esophageal squamous cell carcinoma identified by array comparative genomic hybridization. Genes Chromosom. Cancer.

[27] Kim S., Lee Y., Koo J.S. (2015). Differential expression of lipid metabolism-related proteins in different breast cancer subtypes. PLoS ONE.

[28] Nieman K.M., Kenny H.A., Penicka C.V., Ladanyi A., Buell-Gutbrod R., Zillhardt M.R., Romero I.L., Carey M.S., Mills G.B., Hotamisligil G.S. (2011). Adipocytes promote ovarian cancer metastasis and provide energy for rapid tumor growth. Nat. Med..

[29] Pacilli A., Calienni M., Margarucci S., D’Apolito M., Petillo O., Rocchi L., Pasquinelli G., Nicolai R., Koverech A., Calvani M. (2013). Carnitine-acyltransferase system inhibition, cancer cell death, and prevention of myc-induced lymphomagenesis. J. Natl. Cancer Inst..

[30] Ricciardi M.R., Mirabilii S., Allegretti M., Licchetta R., Calarco A., Torrisi M.R., Foa R., Nicolai R., Peluso G., Tafuri A. (2015). Targeting the leukemia cell metabolism by the CPT1A inhibition: Functional preclinical effects in leukemias. Blood.

[31] Achten J., Jeukendrup A.E. (2004). Optimizing fat oxidation through exercise and diet. Nutrition.

[32] Finelli C., Sommella L., Gioia S., la Sala N., Tarantino G. (2013). Should visceral fat be reduced to increase longevity?. Ageing Res. Rev..

[33] Ewertz M., Jensen M.B., Gunnarsdottir K.A., Hojris I., Jakobsen E.H., Nielsen D., Stenbygaard L.E., Tange U.B., Cold S. (2011). Effect of obesity on prognosis after early-stage breast cancer. J. Clin. Oncol..

[34] Sedlacek S.M., Playdon M.C., Wolfe P., McGinley J.N., Wisthoff M.R., Daeninck E.A., Jiang W., Zhu Z., Thompson H.J. (2011). Effect of a low fat versus a low carbohydrate weight loss dietary intervention on biomarkers of long term survival in breast cancer patients (‘choice’): Study protocol. BMC Cancer.

[35] Adachi Y., Ishiguro J., Kotani H., Hisada T., Ichikawa M., Gondo N., Yoshimura A., Kondo N., Hattori M., Sawaki M. (2016). Comparison of clinical outcomes between luminal invasive ductal carcinoma and luminal invasive lobular carcinoma. BMC Cancer.

[36] Moran M.S., Yang Q., Haffty B.G. (2009). The yale university experience of early-stage invasive lobular carcinoma (ILC) and invasive ductal carcinoma (IDC) treated with breast conservation treatment (BCT): Analysis of clinical-pathologic features, long-term outcomes, and molecular expression of COX-2, BCL-2, and p53 as a function of histology. Breast J..

[37] Garcia-Fernandez A., Lain J.M., Chabrera C., Garcia Font M., Fraile M., Barco I., Torras M., Rene A., Gonzalez S., Gonzalez C. (2015). Comparative long-term study of a large series of patients with invasive ductal carcinoma and invasive lobular carcinoma. Loco-regional recurrence, metastasis, and survival. Breast J..

[38] Orvieto E., Maiorano E., Bottiglieri L., Maisonneuve P., Rotmensz N., Galimberti V., Luini A., Brenelli F., Gatti G., Viale G. (2008). Clinicopathologic characteristics of invasive lobular carcinoma of the breast: Results of an analysis of 530 cases from a single institution. Cancer.

[39] Elston C.W., Ellis I.O. (1991). Pathological prognostic factors in breast cancer. I. The value of histological grade in breast cancer: Experience from a large study with long-term follow-up. Histopathology.

[40] Hammond M.E., Hayes D.F., Dowsett M., Allred D.C., Hagerty K.L., Badve S., Fitzgibbons P.L., Francis G., Goldstein N.S., Hayes M. (2010). American society of clinical oncology/college of american pathologists guideline recommendations for immunohistochemical testing of estrogen and progesterone. J. Clin. Oncol..

[41] Wolff A.C., Hammond M.E., Schwartz J.N., Hagerty K.L., Allred D.C., Cote R.J., Dowsett M., Fitzgibbons P.L., Hanna W.M., Langer A. (2007). American society of clinical oncology/college of american pathologists guideline recommendations for human epidermal growth factor receptor 2 testing in breast cancer. J. Clin. Oncol..

[42] Won K.Y., Kim G.Y., Kim Y.W., Song J.Y., Lim S.J. (2010). Clinicopathologic correlation of beclin-1 and bcl-2 expression in human breast cancer. Hum. Pathol..

[43] Goldhirsch A., Wood W.C., Coates A.S., Gelber R.D., Thurlimann B., Senn H.J. (2011). Strategies for subtypes—Dealing with the diversity of breast cancer: Highlights of the st. Gallen international expert consensus on the primary therapy of early breast cancer 2011. Ann. Oncol..

