# Supplementary material for: Expression of Lipid Metabolism-Related Proteins Differs between Invasive Lobular Carcinoma and Invasive Ductal Carcinoma
Source: Int J Mol Sci. 2017 Jan 23;18(1):232. doi: 10.3390/ijms18010232 (PMC5297861; doi:10.3390/ijms18010232)
Supplement: Supplementary file 1 [file ijms-18-00232-s001.pdf]

Supplementary Materials: Expression of Lipid Metabolism-Related Proteins Differs between Invasive Lobular Carcinoma and Invasive Ductal Carcinoma

Yoon Jin Cha, Hye Min Kim and Ja Seung Koo

Table S1. Clinicopathologic characteristics of invasive lobular carcinoma.

Parameters	Total *n* = 108 (%)	Classic Type *n* = 97 (%)	Pleomorphic Type *n* = 11 (%)	*p*-Value
Age (years)				**0.011**
<50	60 (55.6)	58 (59.8)	2 (18.2)	
≥50	48 (44.4)	39 (40.2)	9 (81.8)	
Nuclear grade				**<0.001**
1/2	97 (89.8)	97 (100.0)	0 (0.0)	
3	11 (10.2)	0 (0.0)	11 (100.0)	
Histologic grade				**<0.001**
I/II	104 (96.3)	97 (100.0)	7 (63.6)	
III	4 (3.7)	0 (0.0)	4 (36.4)	
Pathologic T stage				**0.048**
T1	64 (59.3)	61 (62.9)	3 (27.3)	
T2/T3	44 (40.7)	36 (37.1)	8 (72.7)	
Lymph node metastasis				0.733
Absent	75 (69.4)	68 (70.1)	7 (63.6)	
Present	33 (30.6)	29 (29.9)	4 (36.4)	
ER				0.484
Negative	6 (5.6)	5 (5.2)	1 (9.1)	
Positive	102 (94.4)	92 (94.8)	10 (90.9)	
PR				**0.018**
Negative	18 (16.7)	13 (13.4)	5 (45.5)	
Positive	90 (83.3)	84 (86.6)	6 (54.5)	
HER-2				**0.002**
Negative	101 (93.5)	94 (96.9)	7 (63.6)	
Positive	7 (6.5)	3 (3.1)	4 (36.4)	
Ki-67 LI				**0.001**
≤14	88 (81.5)	84 (86.6)	4 (36.4)	
>14	20 (18.5)	13 (13.4)	7 (63.6)	
Molecular type				**<0.001**
Luminal A	82 (75.9)	79 (81.4)	3 (27.3)	
Luminal B	21 (19.4)	14 (14.4)	7 (63.6)	
HER-2	1 (0.9)	0 (0.0)	1 (9.1)	
TNBC	4 (3.7)	4 (4.1)	0 (0.0)	
Body mass index * (BMI, mean ± SD)	22.9 ± 2.9	22.8 ± 2.9	23.2 ± 2.0	0.697
Tumor recurrence	4 (3.7)	3 (3.1)	1 (9.1)	0.318
No. of patient deaths	5 (4.6)	2 (2.1)	3 (27.3)	0.007
Duration of clinical follow-up (months, mean ± SD)	71.4 ± 27.3	71.7 ± 27.4	69.5 ± 27.7	0.806

ER, estrogen receptor; PR, progesterone receptor; HER-2, human epidermal growth factor receptor 2; LI, labeling index; TNBC, triple-negative breast cancer; SD, standard deviation. * BMI data was not available in three patients. Significant values in bold.

Table S2. Clinicopathologic characteristics of invasive ductal carcinoma.

Parameters	Total (*n* = 476) (%)	Luminal A (*n* = 242) (%)	Luminal B (*n* = 134) (%)	HER-2 (*n* = 50) (%)	TNBC (*n* = 50) (%)	*p*-Value
Age (years)						**<0.001**
<50	250 (52.5)	125 (51.7)	88 (65.7)	18 (36.0)	19 (38.0)	
≥50	226 (47.5)	117 (48.3)	46 (34.3)	32 (64.0)	31 (62.0)	
Histologic grade						**<0.001**
I/II	352 (73.9)	220 (90.9)	87 (64.9)	29 (58.0)	16 (32.0)	
III	124 (26.1)	22 (9.1)	47 (35.1)	21 (42.0)	34 (68.0)	
T stage						0.577
T1	272 (57.6)	145 (59.9)	77 (57.5)	27 (54.0)	25 (50.0)	
T2/T3	202 (42.4)	97 (40.1)	57 (42.5)	23 (46.0)	25 (50.0)	
Lymph node metastasis						0.288
Absent	283 (59.5)	139 (57.4)	78 (58.2)	30 (60.0)	36 (72.0)	
Present	193 (40.5)	103 (42.6)	56 (41.8)	20 (40.0)	14 (28.0)	
ER						**<0.001**
Negative	110 (23.1)	5 (2.1)	5 (3.7)	50 (100.0)	50 (100.0)	
Positive	366 (76.9)	237 (97.6)	129 (96.3)	0 (0.0)	0 (0.0)	
PR						**<0.001**
Negative	162 (34.0)	34 (14.0)	28 (20.9)	50 (100.0)	50 (100.0)	
Positive	314 (66.0)	208 (86.0)	106 (79.1)	0 (0.0)	0 (0.0)	
HER-2						**<0.001**
Negative	349 (73.3)	242 (100.0)	57 (42.5)	0 (0.0)	50 (100.0)	
Positive	127 (26.7)	0 (0.0)	77 (57.5)	50 (100.0)	0 (0.0)	
Ki-67 LI						**<0.001**
≤14	305 (64.1)	242 (100.0)	41 (30.6)	16 (32.0)	6 (12.0)	
>14	171 (35.9)	0 (0.0)	93 (69.4)	34 (68.0)	44 (88.0)	
Body mass index * (BMI, mean ± SD)	25.0 ± 3.6	26.5 ± 3.7	23.4 ± 3.2	23.6 ± 3.2	23.7 ± 3.0	0.753
Tumor recurrence	22 (4.6)	8 (3.3)	5 (3.7)	5 (10.0)	4 (8.0)	0.124
No. of patient deaths	26 (5.5)	9 (3.7)	5 (3.7)	5 (10.0)	7 (14.0)	**0.010**
Duration of clinical follow-up (months, mean ± SD)	58.5 ± 15.3	60.1 ± 14.4	58.8 ± 15.7	52.9 ± 20.0	55.5 ± 13.1	**0.010**

ER, estrogen receptor; PR, progesterone receptor; LI, labeling index; TNBC, triple-negative breast cancer; SD, standard deviation. * BMI data was not available in 168 patients. Significant values in bold.

Table S3. Multivariate cox proportional hazards analysis of disease-free survival and overall survival patients with invasive lobular carcinoma.

Parameters	Disease Free Survival		Overall Survival	
	HR (95% CI)	*p*-Value	HR (95% CI)	*p*-Value
Tumor stage		0.383		0.944
T1	Reference		Reference	
T2/3	7.939 (0.075–835.3)		48987 (0.000–2.709 × 10^{136})	
Lymph node metastasis		0.785		0.936
Absent	Reference		Reference	
Present	0.785 (0.022–28.31)		64851 (0.000–4.198 × 10^{122})	
ER		0.579		0.998
Negative	Reference		Reference	
Positive	0.252 (0.002–32.72)		2.512 (0.000–1.182 × 10^{277})	
PR		0.427		0.959
Negative	Reference		Reference	
Positive	43.850 (0.360–5343)		0.000 (0.000–2.517 × 10^{148})	
Ki-67 LI (%)		0.427		0.331
≤14	Reference		Reference	
>14	3.956 (0.133–117.4)		2.955 (0.332–26.30)	
CPT-1		0.505		0.966
Negative	Reference		Reference	
Positive	3.944 (0.069–223.9)		0.000 (0.000–3.349 × 10^{218})	
Acyl-CoA oxidase 1		0.445		0.940
Negative	Reference		Reference	
Positive	4.184 (0.106–165.1)		1.101 (0.088–13.72)	

HR, hazard ratio; CI, confidence interval; ER, estrogen receptor; PR, progesterone receptor; LI, labeling index; CPT-1, carnitine palmitoyltransferase-1.
